# The promise, problems, and pitfalls of including pregnant women in clinical trials of Lassa fever vaccine: a qualitative assessment of sub-Sahara Africa investigators’ perception

**DOI:** 10.11604/pamj.2022.41.242.33863

**Published:** 2022-03-23

**Authors:** Kolawole Akeem Salami, Henshaw Eyambe Mandi, Nathalie Imbault, Nadia Gabriela Tornieporth

**Affiliations:** 1 World Health Organization, Geneva, Switzerland,; 2 Coalition for Epidemic Preparedness and Innovations, Oslo, Norway,; 3 Coalition for Epidemic Preparedness and Innovations, London, United Kingdom

**Keywords:** Lassa fever, vaccine, clinical trial, pregnancy

## Abstract

**Introduction:**

Lassa fever runs a uniquely severe course in pregnancy. There are plans for Lassa fever vaccine clinical trials in endemic West African countries. We assessed the perception of West African investigators to include pregnant women in these studies.

**Methods:**

interviews were conducted with eight sub-Saharan African investigators. These investigators, listed as speakers at the 9^th^ European and developing countries clinical trials partnership (EDCTP) congress and had clinical research experience in sub-Saharan Africa, were purposefully included as study participants. Six are from West Africa. The information was analyzed thematically.

**Results:**

we interviewed eight (six in-person and two on the phone) out of fifteen earmarked investigators. Respondents had limited experience with pregnant women in clinical trials, but desired a paradigm shift. They identified pregnant women's willingness, a robust community engagement strategy, and adequate safety data as enablers, while lack of safety data, persistent fears about potential harm to pregnant women and offspring, and inappropriate community engagement activities as potential barriers.

**Conclusion:**

the inclusion of pregnant women in Lassa fever vaccine clinical trials should be a priority of vaccine developers. Investigators are willing to conduct these studies provided adequate measures to ensure safety is in place.

## Introduction

Traditionally, pregnant women and their offspring have been excluded from clinical trials designed to ascertain new vaccines and therapeutic agents' safety and efficacy. They are considered a 'vulnerable population' that requires special protection from pharmacological or biological agents with unverified potential adverse effects [1]. However, emerging facts have suggested that the continued exclusion of pregnant women from vaccines and drugs clinical evaluation may be an overlooked form of inequity. This population is especially vulnerable to infectious diseases like malaria, hepatitis E, and influenza. The chances of death from hepatitis E virus infection are significantly higher in pregnant women than non-pregnant adults. Yet, the only vaccine licensed for preventing the disease (Hercolin®) is not indicated for use in pregnant women [2]. The world currently suffers from a COVID-19 pandemic. Pregnant women appeared to have a significantly higher risk for severe COVID-19-associated outcomes than non-pregnant women [3]. Despite this, pregnant and lactating women worldwide appear not to be priorities in the race to bring vaccine candidates to licensure. Pregnancy is also known to complicate the clinical course and worsen the prognosis of women suffering from several emerging and re-emerging infectious diseases (EID). Emerging infectious diseases remain a significant global public health threat, mainly where pathogens are endemic and populations remain susceptible. In the West Africa sub-region, emerging and re-emerging viral infectious diseases with hemorrhagic components like Ebola (EVD), yellow fever (YF), and Lassa fever (LF) constitute serious threats [4,5]. These outbreaks have demonstrated how infectious disease outbreaks can severely affect pregnant women and their offspring's health and well-being. Hence, further underlining the critical need to proactively consider pregnant women and their offspring in vaccine research and development efforts to combat these EIDs [4].

Thanks to extensive north-south global health collaboration, a vaccine for EVD prevention is now licensed for human use [6]. However, the rVSV-EBOV (Ervebo®) vaccine was not evaluated in pregnant women during clinical development; hence it is not indicated for pregnant and lactating women [7]. Nevertheless, more than a thousand pregnant women in the Democratic Republic of Congo (DRC) received the vaccine under “expanded access” or “compassionate use” protocol, during the recent EVD outbreak in the North Kivu region, despite the lack of data supporting its safety or efficacy in this [7,8]. Therefore, the decision to administer the vaccine was based on the benefit-risk appraisal of health authorities. The case of the other vaccine candidate (Ad26.ZEBOV/MVA-BN), now licensed by the European Union (EU), is no different [9,10]. However, there seem to be recent indications of commitments towards addressing this gap [11]. Maternal immunization has emerged as a novel public health tool to reduce neonatal mortality from diseases such as tetanus, respiratory syncytial virus (RSV), and Group B streptococcus sepsis (GBS). Also, the public health importance of diseases such as Zika and Rubella stems from their impact when first acquired in pregnancy. A new approach to vaccine development that takes pregnant women's needs and rights into consideration is a public health priority. In response to this need, regulatory bodies such as the United States Food and Drug Administration (FDA) published guidelines for the clinical testing of investigational agents in pregnant women in 2018 [12]. Groups dedicated to the ethically responsible, socially just, and respectful inclusion of pregnant women's interests in developing and deploying vaccines against emerging pathogens have arisen. One such group is the Pregnancy Research Ethics for Vaccines Epidemics, and new technologies (PREVENT) working group. In consultation with external experts and key stakeholders, the PREVENT group published a guidance document in 2019 that proposed twenty-two recommendations that could serve as a roadmap for pregnant women in evaluating vaccine candidates for EIDs [4].

The year 2020 saw the publication of critical obstetric assessment criteria for including or excluding pregnant women in maternal immunization trials [13]. However, whether these developments would increase the number of vaccine research studies involving pregnant women remains to be seen. Awareness of LF as a disease of global health security importance is growing, and the development of a vaccine for its prevention is a public health priority [14,15]. Lassa fever runs a more severe and often fatal course in pregnancy, where the case fatality rate (CFR) can be as high as 80%, especially in the third trimester of pregnancy. Fetuses fare much worse as LF may lead to a 100% fetal demise, and the fetus's evacuation was observed to improve pregnant women's prognosis [16]. Financial support from organizations such as the Coalition for Epidemic Preparedness Innovations (CEPI) has led to a relatively robust virus Lassa (LASV) vaccine candidate pipeline, with two candidates already in human trials [17,18]. European and Developing countries Clinical Trial Partnership (EDCTP) has also pledged financial support and issued a joint call with CEPI for proposals for the phase 2/3 clinical evaluation of LF vaccine candidates in endemic countries [19]. Therefore, it is trite to discuss a pathway for pregnant women in these vaccine clinical trials. Based on the disease's epidemiology and prevalence in West Africa, vaccine clinical trials are expected to be conducted in the subregion. Therefore, it is vital to determine West African investigators' perceptions of pregnant women's inclusion in LASV vaccine clinical trials and document the enablers and potential barriers.

## Methods

**Study design:** given the explorative nature of the research questions and the limited understanding of the factors responsible for the reluctance in including pregnant women in vaccine clinical trials despite the publication of guidelines [4,20] and enhanced advocacy, we, therefore, adopt a qualitative approach utilizing the participatory research (action inquiry) paradigm to answer the research questions. Data collection occurred between 17-21 September 2018 at the ninth EDCTP Forum in Lisbon, Portugal.

**Respondents:** we used a purposive sampling strategy to guide the selection of “information-rich” participants who have experience in conducting clinical research in sub-Saharan Africa among the researchers listed as speakers at the 9^th^ EDCTP congress. Potential respondents were also identified through an ongoing online site assessment exercise [21]. Core members of the research team discussed the appropriateness of identified likely respondents. Fifteen participants were recruited with the following nationalities: Benin, Burkina Faso, Ghana, Mali, Guinea, Nigeria, and Sierra Leone.

**Data collection:** we conducted six individual in-depth interviews (IDI) with investigators from Burkina Faso (2), Senegal (1), Ghana (1), Mali (1), Sierra (1), and Gabon (1), and Congo-Brazzaville (1) that attended the ninth EDCTP forum. Besides, we conducted two interviews by phone with identified investigators from Sierra Leone and Nigeria. The respondents were clinically qualified scientists with many years of experience in conducting clinical trials. All interviews were conducted in English by the first author. Interview length ranged from 30 to 60 minutes. On average, the face-to-face interviews were longer than the phone interviews by 15 minutes. A semi-structured interview guide was developed and used. The main objectives were; a) to describe the experience of these investigators with pregnant women in clinical trials involving investigational agents while highlighting; b) the enablers and c) barriers to the inclusion of pregnant women in future LASV vaccine clinical trials. Under these broad objectives, we used a flexible and iterative approach to questioning to elicit in-depth information and ensure that relevant issues were not neglected. Data was recorded by audiotape to ensure that the raw data of each interview was captured.

**Data analysis:** recorded IDIs were transcribed to obtain each interview's written records and then analyzed. The researcher personally transcribed the verbatim quotes from the audio recordings of each interview into text. Content analysis of the transcribed data was done to identify key themes. This process involved carefully reading each transcript for content, with interesting quotes highlighted and put in a column on each transcript. We also created another column with more interesting and shorter quotes. A comparison of the highlighted text and the shorter quotes was made through all the transcripts, such that emerging patterns were identified, coded, and analyzed by clustering them into themes and subthemes. This thematic content analysis was done using the Braun and Clarke 6 step inductive analysis technique [22]. It involved an initial familiarization with and immersion in the data, followed by generating initial codes from patterns in the data. We then sought to understand the relationship between these codes to form initial themes. We reviewed these themes to ensure mutual exclusivity and that they are reflective of the data set. We then further defined themes by analyzing the data contained within each theme, following which we produced a report which includes the final analysis.

**Ethics approval and consent to participate:** all methods were carried out in accordance with relevant guidelines and regulations. No ethical approval was required as the study did not involve any confidential data involving human subjects. Written consent was, however, obtained from interviewed participants. They also consented to the recording, archiving and publication of the outcome of the exercise.

## Results

We earmarked fifteen investigators from West Africa for interviews. However, only four of the five respondents at the EDCTP forum agreed. Of the remaining ten absentees, two agreed to phone interviews. We also interviewed two experienced investigators from Central Africa who were keen and approached the researcher in Lisbon. Therefore, in total, we interviewed six investigators in-person and two on the phone. Interviewed participants had between 6 and 25 years of clinical research experience with a median of 12 years. Three themes emerged from the data analysis. These were; 1) “experience of sub-Sahara Africa investigators with pregnant women in clinical trials involving investigational agents,”; 2) “barriers to the inclusion of pregnant women in LASV vaccine clinical trials,” and (2) “the enablers of including pregnant women in LASV vaccine clinical trials.”

**Experience of sub-Sahara Africa investigators with pregnant women in clinical trials involving investigational agents:** the responses revealed that three of the study participants have engaged in clinical trials that involved pregnant women, and most of the experiences with pregnant women were in clinical trials of malaria treatment options in pregnancy. *“I have been involved in a trial of malaria treatment in pregnancy, which occurred in several African countries, and I was declared the investigator for Gabon. So, I had to enroll in that trial about a thousand and two hundred women, pregnant women and administered some drugs that were for the prevention of malaria during pregnancy and follow them up until delivery and follow the children until their first anniversary*" (Participant #9, Investigator, Gabon). *“Yeah, we conducted several studies on pregnant women there is a one study ongoing now where we are assessing the prevention of neonatal sepsis in pregnant women, where we recruit pregnant women when they come for delivery, and we give them a treatment to prevent the infection of the baby. And, we also conducted a few years ago the biggest clinical trial in malaria treatment in pregnant women, where we tested four drugs in pregnant women and recruited about 850 pregnant women in Nanoro for clinical trials. Before that, we also conducted a PK study in pregnant..*. (Participant #2, Clinical Investigator, Burkina Faso). *“Err, clinical trials in pregnant women? only once”* (Participant #1, Investigator, Senegal).

The investigators are mostly willing to include pregnant women in LF vaccine clinical trials. *"...for me, if it's important when you want to test a vaccine against an epidemic disease, to include everybody because when the epidemic comes, it will not make a difference between the study groups"*. (Participant #5, Investigator, Ghana). *“Clinical vaccine trial in pregnant women can be done in a carefully controlled and regulated environment, maybe with a higher level of safety monitoring and evaluation than other groups, I think this is possible, and I believe it has been done in some drugs, and this can be extended to vaccine trials too*. (Participant #6, Investigator, Mali) *“as far as the vaccine is tested in phase 1 and phase 2 trials and we have robust data to convince the ethics committee and the regulatory authorities that we are not exposing the pregnant women at a higher risk by including them in the study...as far as we have enough data to convince them, I think there will not be any problem to include pregnant women in the study”* (Participant #3, Investigator, Sierra Leone). *“Looking at it critically, their continued exclusion cannot be scientifically justified even ethically; it is not acceptable because you are excluding a population that is underserved in clinical trials” (Participant #8, Investigator, Congo). “Although, they are a vulnerable group, with a lot of specific physiologic needs that are different from the nonpregnant populations. It is still paramount that we study them to be able to collect adequate evidence that will be useful when these vaccines are available to the whole population”* (Participant #4, Investigator, Nigeria).

However, two of the investigators expressed their reluctance to include pregnant women in a future LF vaccine clinical trial due to a desire to ‘protect them´ and the risk of reputational damage if maternal deaths or teratogenicity occur. *“yes, it is risky in pregnant women because they are usually easily immunocompromised. And because of that, we don´t want to expose them, and again the possibility of teratogenicity for either candidate vaccine or drugs that you have not proven their safety margin and efficacy. I won´t be keen to conduct such a study” (Participant #7, Investigator, Burkina Faso). “or the ethics committee may look at this scientist (proposing a study of vaccine efficacy in pregnant women) as chasing personal gain as a scientist by willing to put pregnant women at risk so to speak. So, nobody wants to have a bad name” (Participant #5 Investigator Ghana)*.

The responses indicated that pregnant women seemed to have a keen interest in participating in these clinical trials. Most of the investigators believed that everyone, including pregnant women, should be included in clinical trials. *“Even, pregnant women are keener to participate in the study because they think of the baby that the investigator will take care of them better than the normal care provided by the government” and “for me if it's important when you want to test a vaccine against an epidemic disease, to include everybody because when the epidemic comes, it will not make a difference between the study groups”*. (Participant #5, Investigator, Ghana). *“I told you at the beginning that I am involved in coordinating an Ebola vaccine trial in Sierra Leone, and we included in our age groups one of the exclusion criteria was that they should not be pregnant at the time of training, so we conduct pregnancy test for them. You need to see the level of emotional disturbance that most of these prospective participants express when they find out that they are excluded on the basis of being pregnant”* (Participant #3, Investigator, Sierra Leone).

Some of the participants expressed the view that when excluded from clinical trials, pregnant women show some level of emotional disturbance. *"for me, if it's important when you want to test a vaccine against an epidemic disease, to include everybody because when the epidemic comes, it will not make a difference between the study groups"*. (Participant #5, Investigator, Ghana).

**Barriers to the inclusion of pregnant women in LASV vaccine clinical trials:** participants generally explained that for clinical trials involving investigational new drugs (INDs), the usual recruitment criteria are that female subjects are not pregnant and consent to a pregnancy preventive measure during the clinical trial duration. *“so that´s why one of the inclusion criteria in many of the vaccine trials in which I have been involved is that the woman must undergo a pregnancy test before being enrolled, and when they are enrolled, there will be a period of mandatory contraception, i.e., they will be on any form of contraception that is acceptable to the woman to ensure that the woman does not conceive during the period of active participation in the vaccine trial.”* (Participant #3, Investigator, Sierra Leone). Other barriers to pregnant women participating in clinical trials were identified as.

**Inadequate safety information:** *“so this study will be very crucial because first, you need to prove the non-teratogenicity of this vaccine in the animal model and also during this childbearing age women, follow them up until they get pregnant to see at the earlier stage of the pregnancy to see if when they get pregnant, this vaccine is still effective. But during pregnancy, it will be a bit difficult if there is no proof of non-teratogenicity of the drug.”* (Participant #1, Investigator, Senegal).

**Ethical concerns- especially anticipated difficulties in obtaining ethical approval and the competence of ethics committee members in developing countries in reviewing such protocols:** *“but the problem will be the ethics committee and the regulatory authorities, to convince them that we are not exposing pregnant women at a higher risk by enrolling them. No, I think this will also depend on the study design because if you design a study with where you want to recruit a huge number of pregnant women, of course, they will say no; but, if you go gradually to say okay, we will start with first, for instance, five or ten pregnant women.”* (Participant #3, Investigator, Sierra Leone). *“Ethical approval might be very challenging, but it is not impossible; it is doable.you should have all the explanation on hand to give to the ethical committee because your main challenge would be the ethical committee and the quality of the ethical committee members.”* (Participant #6, Investigator, Mali). *“Of course, you will need to convince the ethics committees. Usually ethics committee, they´re not very prone to accept clinical trials on venerable populations”* (Participant #1, Investigator, Senegal).

**The reluctance of trial sponsors to include pregnant women in trials:** *“sometimes it´s not the investigator who doesn´t want to do it in most cases but the companies. If the sponsors are not ready to face the extra scrutiny necessary to gain ethics approval or liability, then there is nothing the local investigator can do”* (Participant #7, Investigator, Burkina Faso).

**Appropriate community engagement strategy:** *“the community´s attitude will depend on the sensitization process; where you tell them clearly that if an outbreak comes today in the village, it will not decide if you are pregnant or you are not pregnant, so everybody could be afflicted; hence, it will be good to have an idea how this vaccine can protect the population including pregnant women”* (Participant #2, Clinical Investigator, Burkina Faso). *“When they are not aware of the benefits or risks of the clinical trial, they are afraid, but when they are aware, it is okay. But at the same time, you have to make them aware that if the clinical trial is not done in such population, there would be no data.”* (Participant #2, Investigator, Burkina Faso). *“the problem we might have recruiting is the clinicians who are managing the women, are going to be some barrier because you need to convince them why the safety for the fetus”* (Participant #5, Investigator, Ghana). *“In Africa, to participate in a study, it depends on the culture normally. I think it will depend on the community. The barrier I can face is the community I would use. I would need to explain to the community the importance of the study.”*(Participant #4, Investigator, Nigeria).

## Discussion

Our study revealed that only three participants had clinical trials with pregnant women, and most of these experiences were with malaria treatment. This is not surprising due to the scientific community's well-established reticence to include pregnant women in clinical trials. Two of the respondents were skeptical about conducting these studies due to safety concerns and the risk of reputation damage if serious adverse events would develop in pregnant women and their offspring during the trial. However, all other investigators believed that everyone, including pregnant women, should be included in clinical trials. The aversion to their inclusion was mainly based on the lack of adequate data on pregnant women and fetus's adverse events, a finding that is consistent with the published literature [23]. However, they expressed their readiness to conduct clinical trials of LASV vaccine in pregnant women in their countries, provided that adequate safety information, and a mechanism for enhanced safety monitoring was put in place by the trial sponsors. Therefore, for LASV vaccine clinical evaluation in pregnancy, preliminary safety data from non-pregnant women will be a prerequisite. To further characterize this, participants suggested that such evaluation in pregnant women should only be commenced after safety information had been collected in the 'usual' healthy adult population up to phase 2 clinical trials. Also, having preclinical safety data from reproductive toxicity studies could facilitate ethics and regulatory approvals. Participants suggested that regulatory authorities should incentivize clinical trials in pregnant women, like the Pediatric Investigating Program (PIP) of the European Medicines Agency (EMA) [24]. The findings also corroborated previous studies indicating pregnant women were keen on participating in clinical trials, and when excluded, they showed some level of emotional disturbance.

Pregnant women are fully competent to provide informed consent for themselves and their offspring. We found that presenting convincing data during the consenting process would enable pregnant women to make informed decisions. This should be especially so in LF in pregnancy. The higher risk of maternal and fetal mortality could make pregnant women more motivated to participate, especially those in endemic areas that had witnessed the disease's burden and worse outcomes in pregnancy. Hence, during the study enrollment, the study teams should be well-trained and table convincing data to pregnant women to guide their decision-making. The risks and benefits should be clearly stated in the patient information sheet and informed consent form during the consenting process. This study revealed that community engagement should be detailed and strategic, to avoid resistance to the trial through misinformation. Most importantly, gatekeepers such as traditional heads, religious leaders, and local health officers need to be convinced of the study's aims and the potential benefit for pregnant women. Thus, a useful community and public engagement strategy should inform the community and involve them during the trial's design, planning, and implementation phases. In West Africa, midwives are probably the first contact during antenatal visits for most pregnant women. Therefore, they could be invaluable resource persons to sensitize pregnant women (and communities) about participation in a proposed clinical trial. Given the evidence of Lassa fever's high maternal and fetal mortality and severe disease in this vulnerable population, women unquestionably require a safe and effective Lassa vaccine during pregnancy. Failure to test the safety and efficacy of Lassa vaccine during pregnancy may endanger the health of both women and their fetuses. Enrolling pregnant women in clinical trials in these resource-constrained Lassa fevers endemic areas could provide direct benefits to women and their offspring that are not available outside the research setting. In a nutshell, developing a Lassa fever vaccine that is safe and effective in pregnancy is a major public health ethics issue. The strength of our study lies in its being the first known attempt to describe the perception of investigators in sub-Sahara Africa to including pregnant women in investigational product clinical trials using qualitative method.

**Limitations:** although all attempts were made to ensure a high-quality non-biased study, the study's relevance and completeness were limited by factors such as the small sample size and the non-response of some investigators. We planned to interview fifteen investigators from West Africa but could only interview six from the sub-region and two additional willing clinical researchers from Central Africa. We also conducted two interviews virtually; therefore, the interviewer was not privy to the subtle information relayed through respondents' body language.

## Conclusion

We strongly recommend that, without a clear justification for exclusion, pregnant women should be included in future clinical trials of LF vaccine in West Africa. The factors identified in the study as militating against clinical research in pregnancy are cogent, but not insurmountable. Other factors, such as the limited availability of baseline epidemiologic data on disease burden and maternal and neonatal outcomes during and after pregnancy, are important considerations to note and address [25].

### What is known about this topic

- Pregnant women are uniquely susceptible to Lassa fever and other emerging infectious diseases;
- Pregnant women are routinely left out of clinical trials evaluating the efficacy of new drugs and vaccines;
- There is a growing body of evidence that their continued exclusion constitutes some form of inequity, and there is advocacy for this to be addressed, but the progress is slow.

### What this study adds

- The study highlights the willingness of investigators to conduct LASV vaccine clinical trials in pregnant women provided adequate safety data are available and enhanced safety surveillance is in place;
- The study documents Investigators' belief that pregnant women are eager to participate in clinical trials and provide informed consent for themselves and their children if presented with convincing data.
